# Exosomes derived from mesenchymal stem cells reverse EMT via TGF-β1/Smad pathway and promote repair of damaged endometrium

**DOI:** 10.1186/s13287-019-1332-8

**Published:** 2019-07-29

**Authors:** Yuan Yao, Ran Chen, Guowu Wang, Yu Zhang, Fang Liu

**Affiliations:** 1 Department of Obstetrics and Gynecology, the First Affiliated Hospital of the Medical College, Shihezi University, No. 107, North Second Road, Shihezi, Xinjiang, 832000 Uygur Autonomous Region China; 2 Department of Gynecology, Suining Central Hospital, No. 127 Desheng West Road, Chuanshan District, Suining, 629000 Sichuan Province China

**Keywords:** Mesenchymal stem cell, Exosomes, Endometrium, Eendometrial epithelial cell, Epithelial-mesenchymal transition, TGF-β/Smad signaling pathway, Intrauterine adhesion

## Abstract

**Background:**

Intrauterine adhesion (IUA) is one of the most serious complications in patients with endometrial repair disorder after injury. Currently, there is no effective treatment for IUA. Stem cell is the main candidate of new therapy, which functions mainly through paracrine mechanism. Stem-derived exosomes (Exo) play an important role in tissue injury. Here, we mainly aim to study the effect of bone marrow mesenchymal stem cell (BMSC)-derived Exo on repairing endometrium of IUA animal models and its effect on TGF-β1 induced EMT in endometrial epithelial cells (EECs).

**Methods:**

Totally, 64 female rabbits were randomly divided into Sham operation group, model group, BMSC treatment group, and Exo treatment group. EMT in EECs was induced by TGF-β1. Then, EECs were treated with Exo (25 μg/ml, 50 μg/ml, 100 μg/ml) for 24 h. HE staining and Masson staining were used to evaluate the changes in glandular number and fibrosis area. The expression levels of CK19 and VIM were detected by immunohistochemistry. Western blotting was used to detect the expression of CK19, VIM, FSP-1, E-cadherin, TGF-β1, TGF-β1R, Smad 2, and P-Smad 2. RT-PCR was used to detect mRNA expression levels of CK19, VIM, FSP-1, E-cadherin, TGF-β1, TGF-β1R, and Smad 2.

**Results:**

Compared with the model group, the number of endometrial glands was significantly increased and endometrial fibrosis area was significantly decreased in BMSC and Exo groups (*P* < 0.05). CK19 level significantly increased whereas VIM level significantly decreased after treatment of BMSCs and Exo (*P* < 0.05). Additionally, the expressions of TGF-β1, TGF-β1R, and Smad2 mRNA were all significantly decreased after BMSC and Exo treatment (*P* < 0.05). Besides, phosphorylation levels of TGF-β1, TGF-β1R, and Smad2 were also significantly decreased in BMSC and Exo treatment groups (*P* < 0.05). Furthermore, there was no significant difference between BMSC and Exo treatment groups (*P* > 0.05). EMT was induced in EECs by 60 ng/ml TGF-β1 for 24 h. After Exo treatment for 24 h, mRNA expressions of CK-19 and E-cadherin increased, while those of VIM, FSP-1, TGF-β1, and Smad2 decreased. Additionally, protein expressions of CK-19 and E-cadherin increased, while those of VIM, FSP-1, TGF-β1, Smad2, and P-Smad2 decreased.

**Conclusions:**

BMSC-derived Exo is involved in the repair of injured endometrium, with similar effect to that of BMSC, and can reverse EMT in rabbit EECs induced by TGF-β1. BMSC-derived Exo may promote endometrial repair by the TGF-β1/Smad signaling pathway.

## Background

Endometrial repair disorder after injury refers to the damage of endometrial basal layer, which leads to intrauterine adhesion (IUA), amenorrhea, secondary infertility, and other diseases as the result of endometrial fibrous repair [1]. Epithelial-mesenchymal transition (EMT), one of the most important mechanisms of fibrotic diseases [2], has been found to be closely related to the occurrence of IUA presently. For example, our previous work found that Vimentin (VIM) was highly expressed in injured endometrial epithelial cell of IUA animal model [3]. Li et al. [4] found that miroRNA-29b could suppress endometrial fibrosis by blocking Sp1-TGF-β1/Smad-CTGF pathway. Salma et al. [5] reported that TGF-β1/Smad3/smad7 was the main pathway involved in IUA. It is suggested that endometrial fibrosis might be the result of EMT in the endometrial cell, which eventually leads to endometrial scar healing.

Mesenchymal stem cells (MSCs) are a kind of adult stem cells with self-replication and multi-directional differentiation potential. Zhang et al. [6] reported that there were MSCs in the endometrium, and their deficiency may be related to the occurrence of IUA. Our previous study found that BMSCs had repair effect on rabbit injured endometrium [7]. Du et al. [8] found that after transplantation of bone marrow mesenchymal stem cells (BMSCs) in vivo, BMSCs migrated to endometrial injury site and differentiated into cells with the decidual phenotype of endometrial stromal cells. Wang et al. [9] proposed that transplantation of BMSCs effectively repaired injured endometrium. These findings demonstrate that MSCs could migrate to the lesion and repair the lesion after its transplantation in vivo. However, colonization of MSCs in the injury site is relatively few and MCSs might also perform a repairing role mainly through its paracrine mechanism [10]. Exosome (Exo), secreted by BMSCs, is about 30–150 nm in diameter and contains active substances involved in cellular communication, such as protein, mRNA, and microRNA. In a mouse model of *E. coli*-induced lung injury, MSCs-derived Exo was found to be able to accelerate lung epithelial repair [11]; BMSC-derived Exo could improve cognitive disorder of streptozotocin-diabetic mice by repairing injured neurons and astrocytes [12]; McBride et al. [13] proposed that BMSC-derived Exo could promote proliferation and migration of fibroblast and endothelial cells, as well as stimulate dermis repair and regeneration through regulating the Wnt signaling pathway. Thus, MSCs-derived Exo also plays an important role in repairing the injured tissues.

Herein, we investigated the effect of BMSC-derived Exo on the endometrium of IUA and on TGF-β1 induced EMT in endometrial epithelial cells (EECs). The underlying mechanism was also analyzed and discussed. Our findings may provide a theoretical basis for the development of potential reagents for the treatment of IUA.

## Materials and methods

### Materials

#### Experimental animals

The 4-week-old female New Zealand white rabbits (1.0–1.5 kg) were used for the isolation of BMSCs. The 12-week-old female New Zealand white rabbits (2.5–3.5 kg) were used for the establishment of the IUA model and the isolation of EECs. All the animals were purchased from the Experimental Animal Center of Xinjiang Medical University. All animal experiments were conducted according to the ethical guidelines of Shihezi University.

### Methods

#### Isolation, identification, and differentiation of BMSCs

BMSCs were isolated from the tibia and femur of 4-week-old rabbits using the method of adherent culture of whole bone marrow. The isolated cells were cultured in DMEM (Gibco, Cat #: 1655697) containing 10% fetal bovine serum, 1% penicillin-streptomycin (Gibco, Cat#: 1631430) at 37 °C, 5% CO2. The medium was changed every 3 days until the confluency reached 80%. The cells of the 3rd passage were used. BMSCs were identified by specific expression of CD44 (Bioss, bs-4916R), CD29 (ab78502, Abcam), CD34 (Bioss, bs-0646R), and CD45 (ab30446, Abcam) on BriCyte E6 (Mindray DS US Inc., NJ, USA) flow cytometer.

Adipocyte induction of BMSCs: when the cells reached 70 to 80% confluency, complete medium was replaced with adipogenic induction medium in the next 3 weeks. Then, Oil-Red-O staining was performed.

Osteoblast induction of BMSCs: when the cell reached 60 to 70% confluency, complete medium was replaced with osteogenic induction medium in the next 3 to 4 weeks. Then, Alizarin-Red staining was performed.

#### Extraction, concentration determination, and identification of Exo

When cell confluency reached 80%, serum-free medium was replaced. After 72 h of culture, the cell culture supernatant was collected, and Exo was extracted using Total Exosome Isolation Reagent (Cat#4478359, Invitrogen, USA). BCA protein quantification kit (Cat# BL521A, Biosharp Co. Ltd., Hefei, China) was used to detect Exo protein content. The morphology of Exo was observed under a transmission electron microscope (HITACHI, H-600). Western blot was used to identify the expression of Exo-specific markers CD9 (ab19761, Abeam) and heat shock protein (HSP) 70 (bs-0126R, Bioss).

#### Animal models

A total of 64 female New Zealand white rabbits aged 10–12 weeks were randomly divided into Sham operation group, model group, BMSC treatment group, and Exo treatment group. The IUA model was established using a previously described double injury method [3]. Briefly, After anesthesia with 3% sodium pentobarbital (1 mL/kg), the laparotomy was performed and the bilateral uterus was exposed. An incision at the bilateral uterus junctions was made with ophthalmic scissors, and the uterus spatula was inserted into the uterine cavity through the incision for scraping until a rough feeling on the uterine wall was felt. LPS-soaked cotton thread was then placed into the uterine cavity through the uterine incision, leaving about 5 cm of cotton thread outside to remove the cotton thread later. The abdominal wounds were stitched. One week after modeling, the laparotomy was performed again. The BMSC treatment group was injected with 5 × 10^5^ BMSC suspensions into the longitudinal axis muscle walls of the uterus on both sides, while the Exo group was injected with 0.25 mL Exo (200 μg/mL) suspension into the longitudinal muscle walls of the uterus on both sides. The other two groups were injected with the same volume of normal saline. After 1 week, 2 weeks, 3 weeks, and 4 weeks of treatment, rabbits were sacrificed and uterine tissues were collected.

#### Hematoxylin-eosin (HE) staining and Masson staining

Paraffin sections of rabbit tissue samples were subjected to HE and Masson staining according to routine procedures. Four high-magnification fields were selected for each HE-stained section, and the number of glands in each field of view was counted and averaged. Four high-magnification fields were selected for each Masson stained slice, and the fibrosis area ratio was calculated as follows: total area of endometrial fibrosis per field/the sum area of endometrial stroma and gland. The rate was automatically averaged using the Image-pro Plus software (Media Cybernetics, Inc., MD, USA).

#### Immunohistochemistry

##### Endometrial tissue

The sections were gradually dehydrated and antigen retrieval was carried out in boiling sodium citrate buffer for 10 min. After washing with PBS, the sections were incubated with 3% H_2_O_2_-PBS buffer for 10 min at room temperature and then rinsed with PBS again. Subsequently, rabbit anti-cytokeratin-19 (CK-19; 1:100; Cat# bs-2190R, Bioss Antibodies, USA) or mouse anti-VIM (1200, Cat# bs-8533R, Boster Biological Technology Co. Ltd., CA, USA) was added and incubated at 4 °C overnight. Secondary antibody (Cat# ZB-2301, ZSGB-BIO, Beijing, China) was then added and incubated at 37 °C for 30 min. Color was developed with DAB (Sigma Co., St. Louis, MO, USA) and counterstained with hematoxylin. The sections were observed under an optical microscope (Eclipse E200, Nikon Co., Tokyo, Japan) and photographed. The protein expressions were analyzed with the Image-pro Plus image analysis software (Media Cybernetics, MD, USA).

#### Isolation and identification of EECs

First 100 IU pregnant mare serum gonadotropin (Solarbio) was given to New Zealand rabbits via intramuscular injection, and then 48 h later, intravenous injection of 80 IU human Chorionic Gonadotropin (Ningbo Sansheng Pharmaceutical Company, S170303) was given via ear vein. After 18 h, the rabbits were sacrificed by air embolism through the ear vein. The uterus was isolated and a longitudinal cut was made. The endometrium was gently scraped until the endometrial surface turned whitish. The scraped tissues were collected, centrifuged, and resuspended in PBS containing 0.05% collagenase I (Sigma) and 0.05% trypsin (Biosharp). After digestion at 37 °C for 30 min, the samples were centrifuged and then filtered with a 150-nm strainer and a 38-nm strainer successively. The samples retained on the 38-nm strainer were defined as EECs. They were collected, re-suspended in DMEM-F12 supplemented with 10% fetal bovine serum, 1% penicillin-streptomycin, 100 nmol/L estradiol, 10 nmol/L progesterone, and 5 μg/L epidermal growth factor (Sigma), and cultured at 37 °C with 5% CO2 and 95% humidity.

#### Treatment of EECs

To induce EMT, rabbit EECs were treated with different concentrations of TGF-β1 (0 ng/ml, 10 ng/ml, 60 ng/ml, and, 110 ng/ml), for 24 h and 48 h. Cell morphology changes were observed and cell apoptosis was detected.

To observe the effect of Exo on EMT, EECs were first treated with 60 ng/ml TGF-β1 for 24 h and then with Exo of different concentrations (0 μg/ml, 25 μg/ml, 50 μg/ml, and 100 μg/ml) for 24 h.

#### Cell apoptosis

After treatment with treated with different concentrations of TGF-β1 for 24 h and 48 h, EECs were collected, washed, and re-suspended in 200 μl of binding buffer. After that, EECs were incubated with Annexin V-FITC at room temperature for 15 min and then with PI at 4 °C for 30 min in the dark. Finally, apoptosis was analyzed with flow cytometry.

#### Western blot

The total protein was extracted, and the protein concentration was determined by BCA method. The proteins were separated on SDS-PAGE and then transferred to PVDF membranes. The membranes were blocked with 5% skim milk. The primary antibodies of anti-TGF-β1 (1:500; Cat# bs-0086R; Bioss Antibodies, USA), anti-TGF-β1R (1:500; Cat# bs-23446R; Bioss Antibodies, USA), anti-smad2 (1:500; Cat# bs-0718R; Bioss Antibodies, USA), anti-p-smad2 (1:500; Cat# bs-3420R; Bioss Antibodies, USA), CK19 (1:500; Bioss, bs-2190), anti-VIM (1:500; Bioss, bs-8533R), anti- Fibroblast-specific protein-1 (FSP-1) (1:500; Bioss, bs-3759R), anti-E-cadherin (1:500; Bioss, bs-10009R) and anti-GAPDH (1:2000; Cat# bsm330033m, Bioss Antibodies, USA) were added and incubated overnight at 4 °C. After washing, secondary antibody (ZB-2301 and ZB-2305, ZSBG-BIO, Beijing, China) was added and incubated at 37 °C for 1 h in the dark. The membrane was developed with ECL. The relative expression was calculated by measuring the gray value of protein band using ImageJ software (National, Institute of Health, USA). Three replicates were performed in each group.

#### Real-time PCR

Total RNA was extracted from tissues and cells using total RNA extraction kit (DP431, Tiangen Biotech (Beijing) Co. Ltd., China), and cDNA was synthesized using the PrimeScript™ RT Reagent Kit (Perfect Real Time) (TAKARA Bio Inc., Shiga, Japan). The target genes were amplified by PCR using the following conditions: pre-denaturation at 95 °C for 2 min, followed by 40 cycles of denaturation at 95 °C for 10 s, annealing and extension at 60 °C for 10 s. Three replicates were performed in each group. The primers were listed in Table 1.

**Table 1:** Primer information

Gene name	Primer name	Sequence (5′ → 3′)
TGF-β1	TGF-β1-F	AACACAGCAGAGTGGTTGTC
	TGF-β1-R	TGTCCAGGCTCCAGATGTA
TGF-β_R_1	TGF-β_R_1-F	GATTTGGAGAAGTTTGGCG
	TGF-β_R_1-R	TCCCAGAATACTAAGCCCAT
Smad2	Smad2-F	CAGGAAGAGAAGTGGTGTGA
	Smad2-R	ATACTGGAGGCAGAACTGGT
CK19	rabbit-CK19-F	GCGAACAGCCACTACTTCA
	rabbit-CK19-R	GTCTCAAACTTGGTTCGGA
VIM	rabbit-VIM-F	CGTTGACAATGCTTCTTTGG
	rabbit-VIM-R	TGGATTTCCTCATCGTGC
FSP-1	rabbit-FSP1-F	GGGAAAGAGGGTGACAAGTT
	rabbit-FSP1-R	GTCCAAGTTGCTCATCAGC
E-cadherin	rabbit-Ecadherin-F	ACCCAGGTCTTCTACAGCAT
	rabbit-Ecadherin-R	GATGTGTTCTCGGTCCAGA
GAPDH	GAPDH-F	CTTTGGTATCGTGGAAGGA
	GAPDH-R	AGGGATGATGTTCTGGAGAG

#### Statistical analysis

The data were analyzed with the statistical software SPSS 19.0 (SPSS Inc., Chicago, IL, USA). The data was expressed as mean ± standard deviation (SD). Two samples were compared using independent sample *t* test. When multiple groups of mean variances were homogeneous, single factor analysis of variance (ANOVA) was used, and the LSD method was used for pairwise comparison between groups. *P* < 0.05 was considered as statistically significant.

## Results

### Identification of BMSCs

**Fig. 1:**
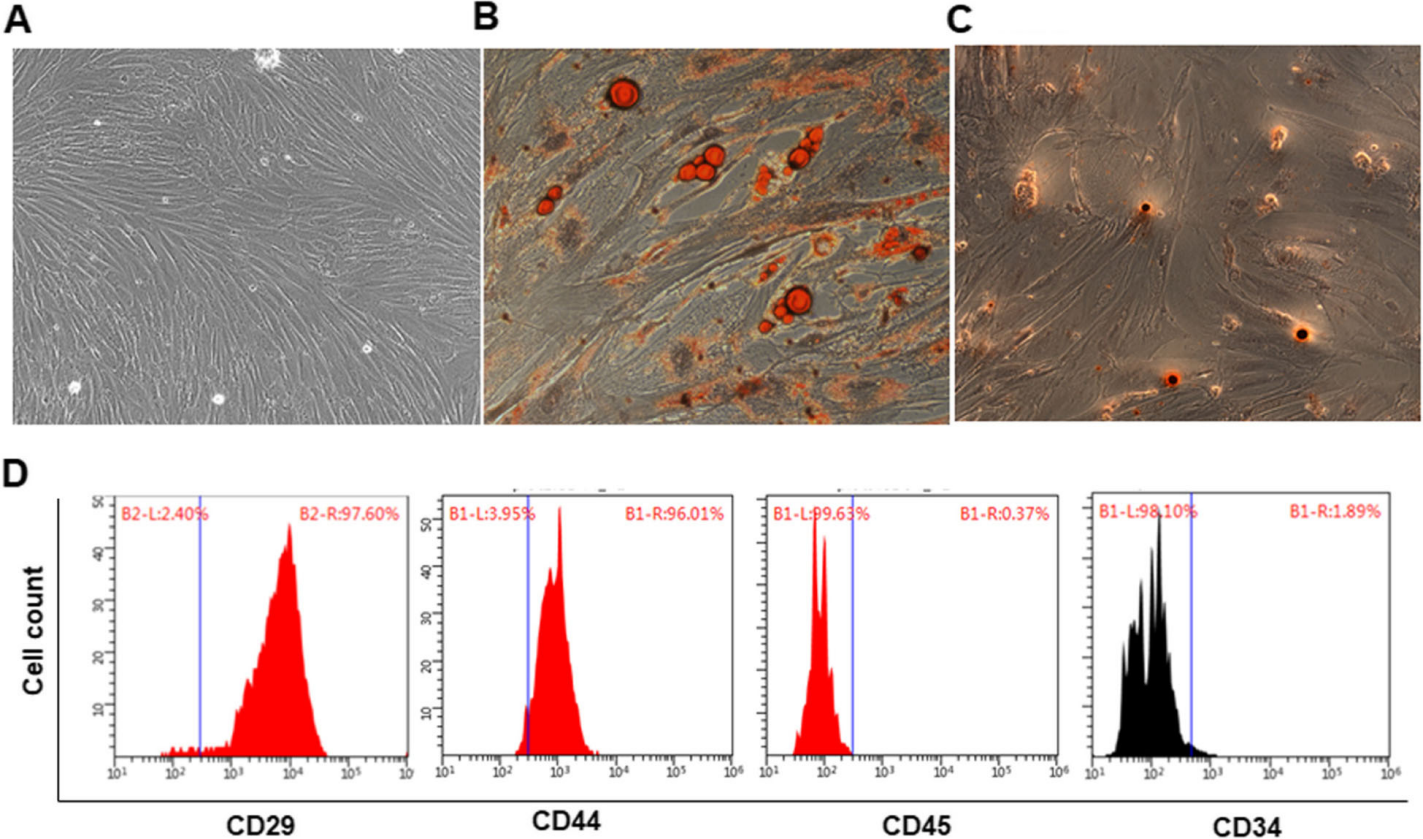
BMSCs and cell morphology after induction of differentiation. **a** P3 generation BMSCs (× 100). **b** Adipogenic induction of BMSCs at 21d. Oil red staining result was shown (× 200). **c** Osteogenic induction of BMSCs for 21d. Alizarin red staining result was shown (× 200). **d** Detection of CD29, CD44, CD45 and CD34 by flow cytometry

After 3 generations of culture, the cells showed fibroblast-like morphology, grew adherently, and arranged in a spiral pattern (Fig. 1a). Adipogenic induction of BMSCs was carried out and the fat cells were identified by Oil Red O staining. As shown in Fig. 1b, vacuoles were observed in the cytoplasm, which were gathered together to form obvious lipid droplets. After osteogenic induction and Alizarin-Red staining of BMSCs, cells showed a nodular shape. There were collagen accumulation and calcium salt precipitation, which formed opaque mineralized nodules showing brownish red after Alizarin-Red staining (Fig. 1c). The results of flow cytometry showed that the surface markers of CD29 (97.60%) and CD44 (96.01%) positive were for BMSCs, and the surface markers of CD45 (1.89%) and CD34 (0.37%) for hematopoietic stem cells were negative (Fig. 1d). These results show that BMSCs are successfully isolated and identified.

### Identification of BMSC-derived Exo

**Fig. 2:**
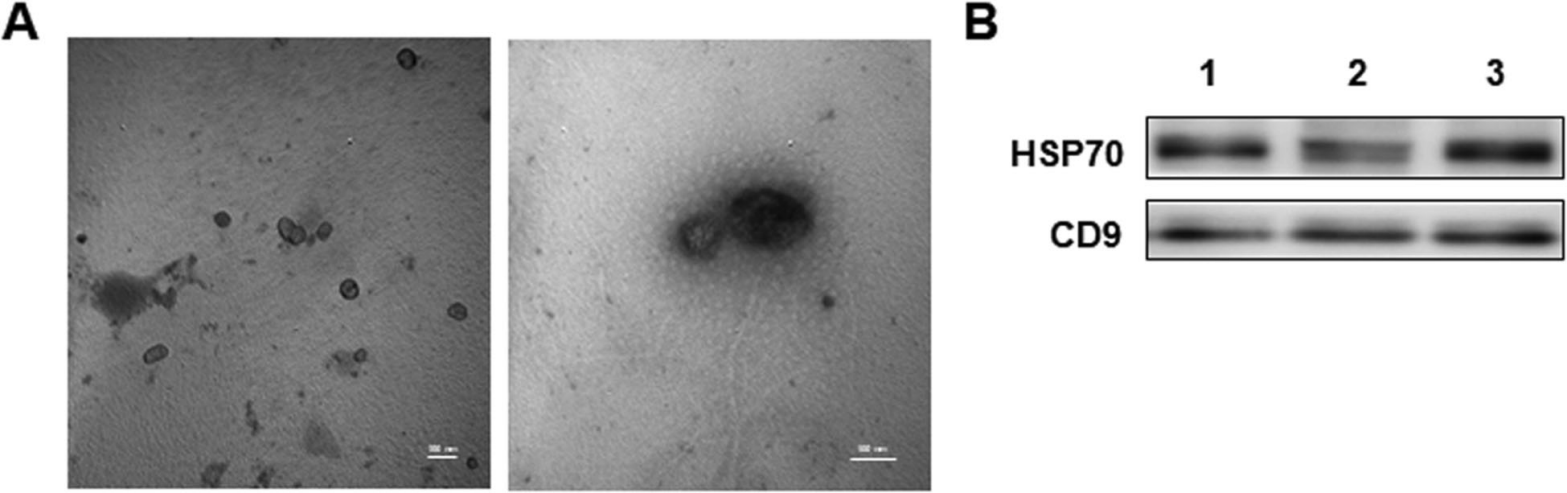
Morphology of Exosomes under electron microscope and expression of its surface marker. **a** The exosomes derived from BMSCs were observed by TEM. **b** Western blot was used to identify the expression of HSP70 and CD9, the specific surface proteins of Exosomes. Lanes 1, 2, and 3 represent the three repeats of exosome sample

The morphology of Exo was observed under a transmission electron microscope. As shown in Fig. 2a, Exo was round or elliptical vesicles with uneven size and intact capsule. It had a diameter of 40–160 nm. The peak particle size was 130 ± 11 nm. The concentration of Exo was determined at 193.6 μg/ml by BCA assay. Western blot was used to detect Exo-specific markers of CD9 and HSP70, and both of them were expressed (Fig. 2b).

### BMSC and Exo treatment increase the number of glands of the endometrial damaged uterus

**Fig. 3:**
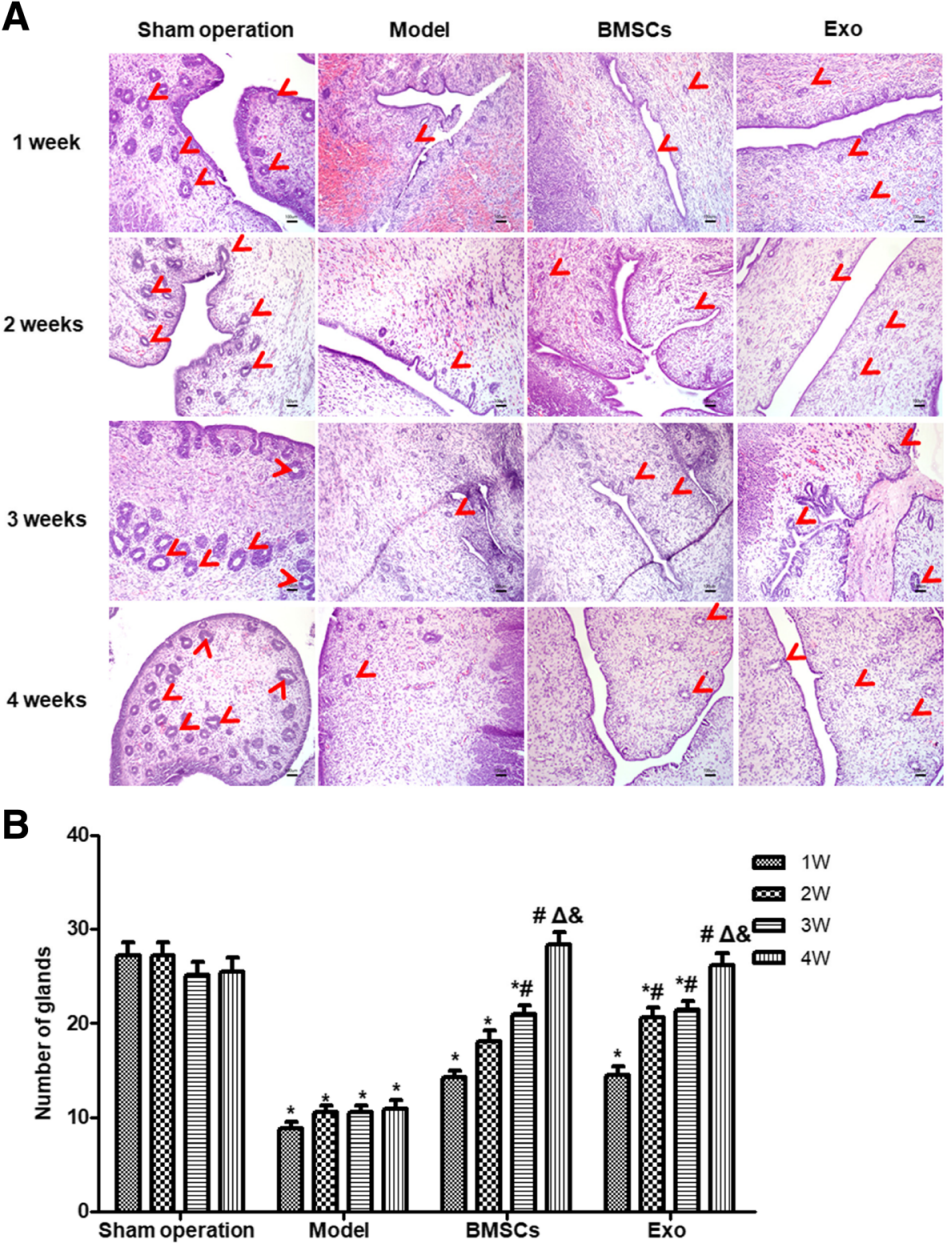
Changes in the number of endometrial glands in rabbit uterus after BMSC and EXO treatment. **a** HE staining was performed to detect endometrial gland changes in each group at different time points. **b** Statistical results of gland number after endometrial damage and treatment. *P < 0.05, compared to the Sham operation group; ^#^*P* < 0.05, compared with 1 week within the same group; ^Δ^*P* < 0.05, compared with 2 weeks within the same group; ^&^*P* < 0.05, compared with 3 weeks within the same group

Rabbit model of IUA was established, and the rabbit uterus tissue was collected at 1st week, 2nd week, 3rd week, and 4th week after modeling. The HE staining results showed that in the Sham operation group, the shape of the uterine cavity of the rabbit was irregular. The surface of the uterine cavity and the glandular cavity were covered with a single layer of columnar epithelium. The epithelial cells were structurally intact, and the interstitial glands were rich and mostly elliptical. In the model group, connective tissue fragments were observed in the uterine cavity after 1 week of damage, and the number of glandular vessels was significantly reduced with connective tissue congestion. The BMSC treatment group and the Exo treatment group showed different degrees of endometrial gland number increase as the treatment time prolonged, and the neonatal duct lumen was not completely covered with a single layer of columnar epithelium (Fig. 3a). Statistical analysis showed that the number of endometrial glands in the model group did not significantly change with prolonged treatment time (*P* > 0.05), while that in the BMSC and Exo groups increased significantly (*P* < 0.05) (Fig. 3b). The numbers of endometrial glands in the model group, BMSC group, and Exo group were significantly lower than that in the Sham operation group (*P* < 0.05) in the first 3 weeks after treatment. In the 4th week after treatment, the number of glands in the model group was significantly lower than that in the Sham operation group (*P* < 0.05), but that of the BMSC group and Exo group had no significant difference from Sham operation group (*P* > 0.05). There was no significant difference between the BMSC group and Exo group (*P* > 0.05). These results suggest that both BMSC and Exo treatment significantly increased the number of endometrial glands.

### BMSC and Exo treatment reduce fibrosis area of the endometrial damaged uterus

**Fig. 4:**
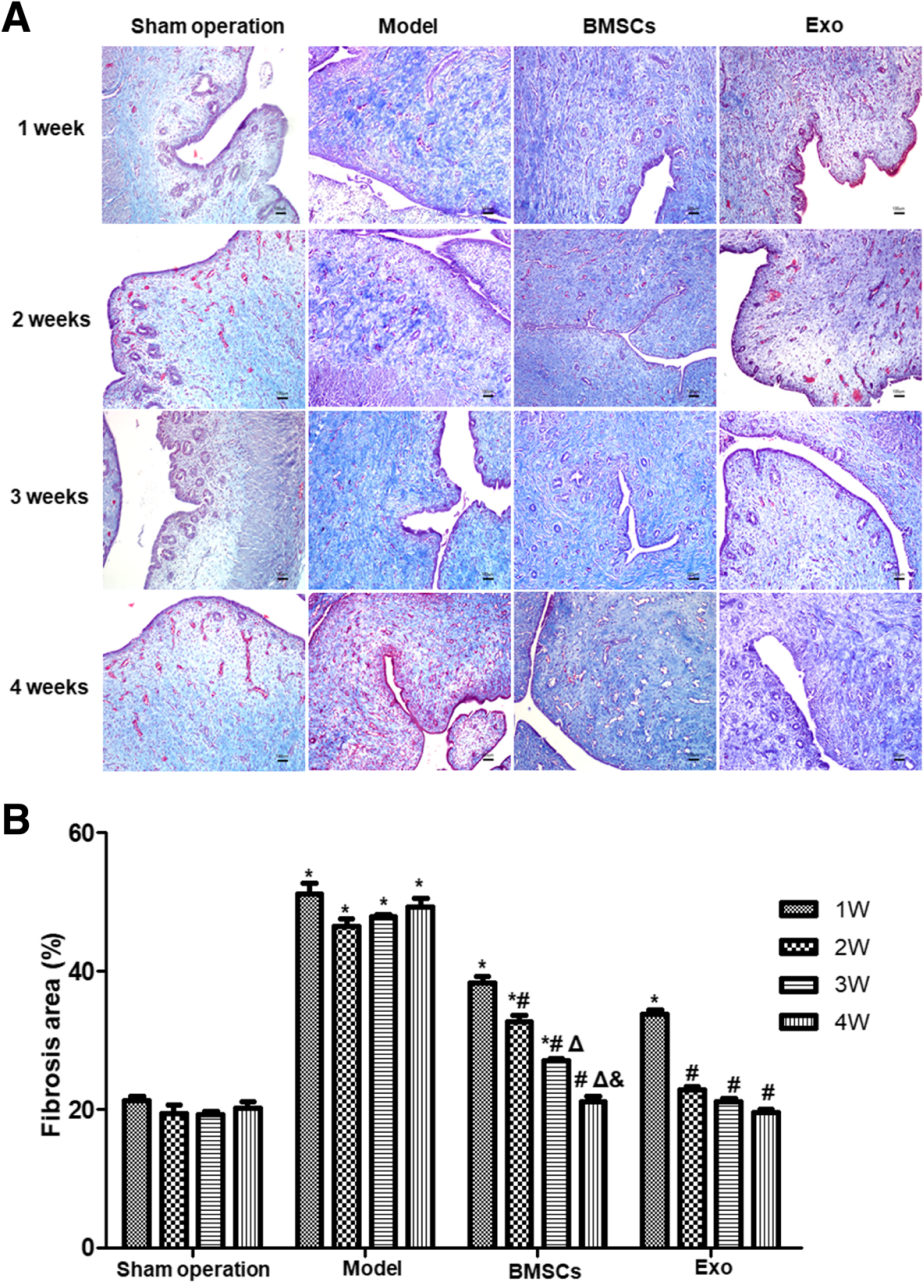
Intrauterine fibrosis of the rabbits after treated with BMSCs and EXO. **a** Masson staining was performed to detect intrauterine fibrosis changes in each group at different time points. **b** Statistical results of intrauterine fibrosis changes after endometrial damage and treatment. *P < 0.05, compared to the Sham operation group; ^#^*P* < 0.05, compared with 1 week within the same group; ^Δ^*P* < 0.05, compared with 2 weeks within the same group; ^&^*P* < 0.05, compared with 3 weeks within the same group

Masson staining showed that in the Sham operation group, the endometrial collagen fibers were blue and arranged neatly, and the mucosa, submucosa, muscles, and blood vessels were red (Fig. 4a). After 1 week of modeling, the collagen fibers of the connective tissue were not uniformly stained. The uterine cavity at the 2nd week, 3rd week, and 4th week after modeling were not obviously changed compared to that at the 1st week of modeling (Fig. 4a), but the collagen fibers in the connective tissue layer were observed, accompanied by an increase in new blood vessels and smooth muscles. In the BMSC and Exo treatment groups (Fig. 4a), the area ratio of blue stained collagen fibers in endometrial mesenchymal decreased from the 1st week after treatment, and the area of fibrosis continued to decrease at the 2nd week after treatment. After 4 weeks of treatment, there was only a small amount of blue collagen fibers in the interstitial region, and they were evenly arranged (Fig. 4a).

Analysis of the endometrial fibrosis showed that the fibrosis area of the Sham operation group was significantly lower than that of the model group at each time point (*P* < 0.05) (Fig. 4b). The area of fibrosis decreased gradually with the treatment time in the Exo group. There was no significant difference in the endometrial fibrosis area from the Sham operation group at 2 weeks after treatment (*P* > 0.05). At 3 weeks and 4 weeks, there was no significant difference between the Exo group and the Sham operation group at each time point (*P* > 0.05). The area of endometrial fibrosis decreased gradually after treatment with BMSCs until the 4th week after treatment, and there was no significant difference compared with the Sham operation group (*P* > 0.05) (Fig. 4b). These results suggest that both BMSC and Exo treatment can reverse endometrial fibrosis.

### BMSC and Exo treatment increase CK-19 expression of the endometrial damaged uterus

**Fig. 5:**
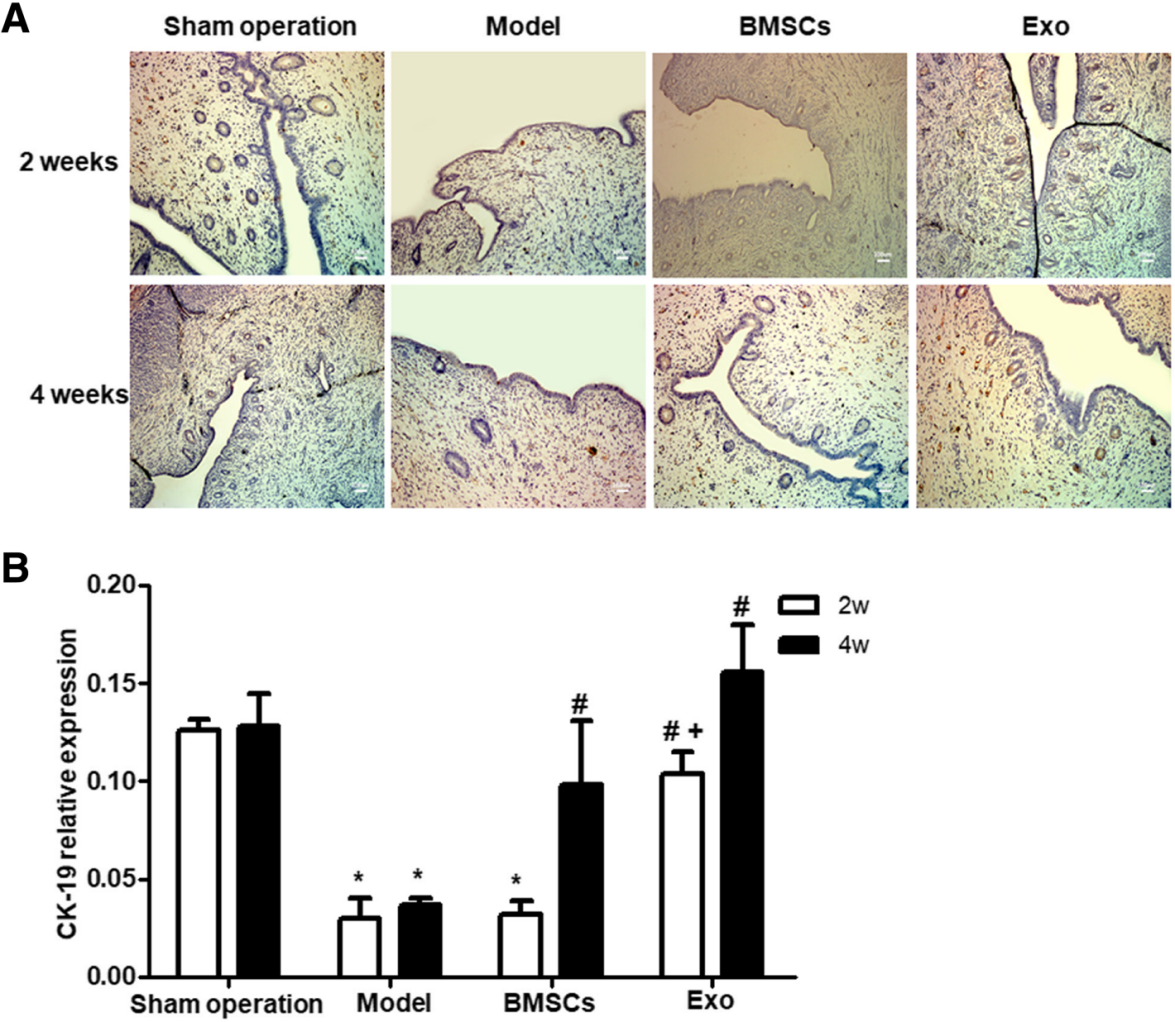
Expression of CK-19 in rabbit endometrium after BMSC and EXO treatment. **a** Immunohistochemical identification of CK19 in the rabbit endometrium. **b** Statistical results of CK19 expression. *P < 0.05, compared to the Sham operation group; ^#^*P* < 0.05 compared to the model group; ^+^*P* < 0.05, comparison between the BMSC and EXO treatment groups

CK19 is a cytoskeletal protein that maintains the integrity of endometrial cells, and its expression is reduced after tissue damage. To determine the expression of CK-19 after uterus damage, immunohistochemical staining of uterine tissue sections was performed. Positive staining of CK-19 protein displayed brown color on cell membrane. The results showed that there was positive staining of CK-19 protein in Sham group, and less positive staining in the model group (Fig. 5a). The expression of CK19 in the BMSC treatment group was not significantly different from that in the Model group at 2 weeks of treatment (*P* > 0.05), but the expression of CK19 was significantly increased after Exo treatment for 2 weeks, compared to the Model group (*P* < 0.05) (Fig. 5b) and there were more brown-yellow particles in the endometrial epithelium. The expression of CK19 in the BMSC treatment group was significantly higher than that in the model group after 4 weeks of treatment (*P* < 0.05), but there was no significant difference between the BMSC treatment group and the Exo treatment group (*P* > 0.05), and many brownish yellow particles were observed in the endometrial epithelium in both groups. These results indicate that both BMSCs and Exo can upregulate the expression of CK19 in cells.

### BMSC and Exo treatment reduce VIM expression of the endometrial damaged uterus

**Fig. 6:**
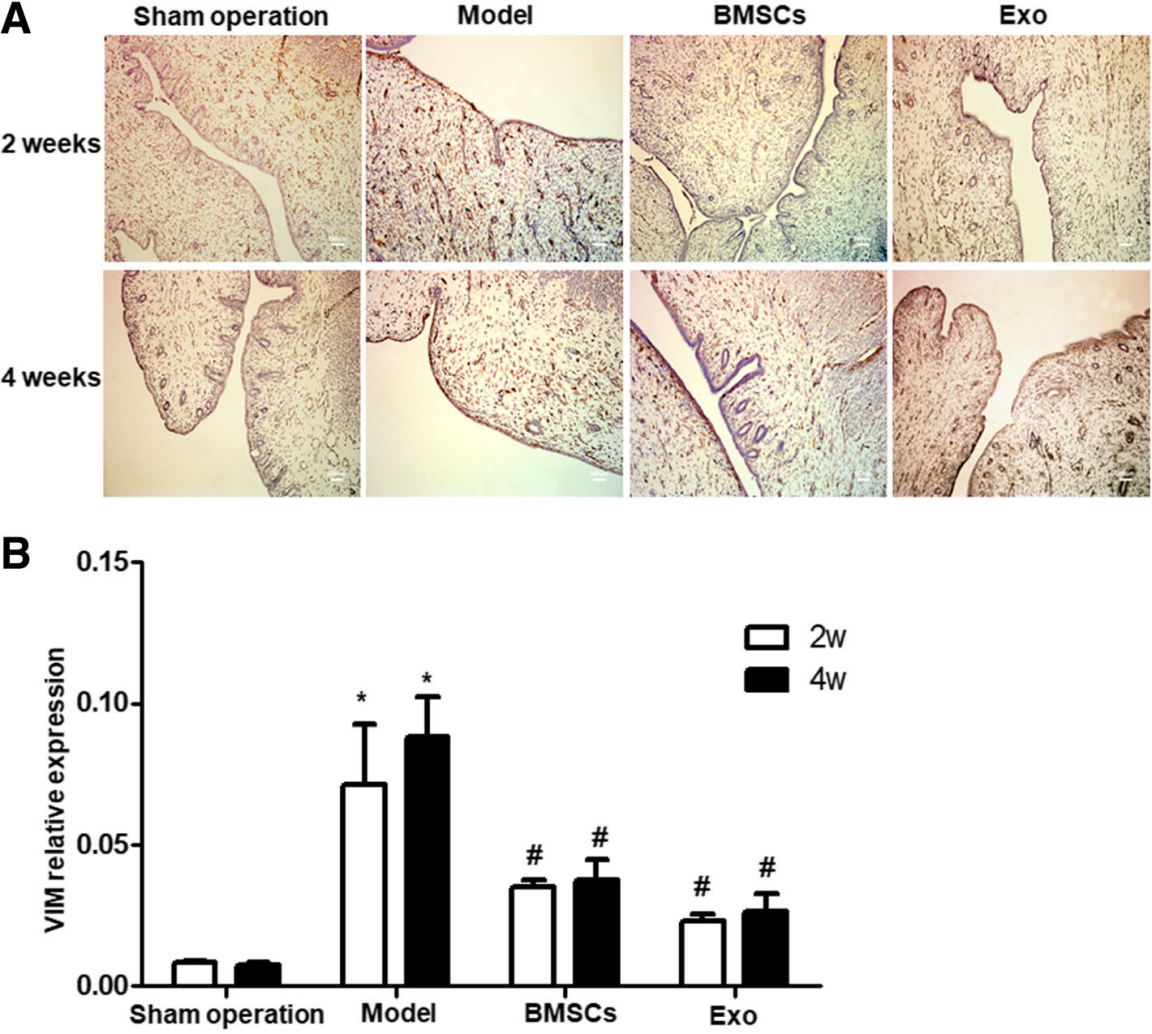
Expression of VIM in rabbit endometrium after BMSC and EXO treatment. **a** Immunohistochemical identification of VIM in the rabbit endometrium. **b** Statistical results of VIM expression *P < 0.05, compared to the Sham operation group; ^#^*P* < 0.05 compared to the model group

Immunohistochemistry of VIM protein showed brown-yellow granules in the endometrial stroma of the rabbits in the Sham operation group, and almost no coloration in the endometrial epithelium. The model group showed brown-yellow particles in the endometrial glandular epithelium, while the BMSC and Exo treatment group showed a small amount of brown-yellow granules in both the endometrial glandular epithelium and the endometrial stroma (Fig. 6a). Statistical analysis showed that VIM expression gradually decreased with prolonged treatment time. After 2 weeks of treatment, there was no significant difference between the BMSC group and the Sham operation group, or between the Exo group and the Sham operation group (*P* > 0.05) (Fig. 6b). In addition, the VIM expression levels in all three groups were significantly lower than that of the model group (*P* < 0.05). These suggest that BMSC and Exo treatment significantly down-regulate the expression of VIM.

### The effect of BMSC and Exo treatment on the mRNA expressions of TGF-β1, TGF-β1R, and Samd2 mRNA of the endometrial damaged uterus

**Fig. 7:**
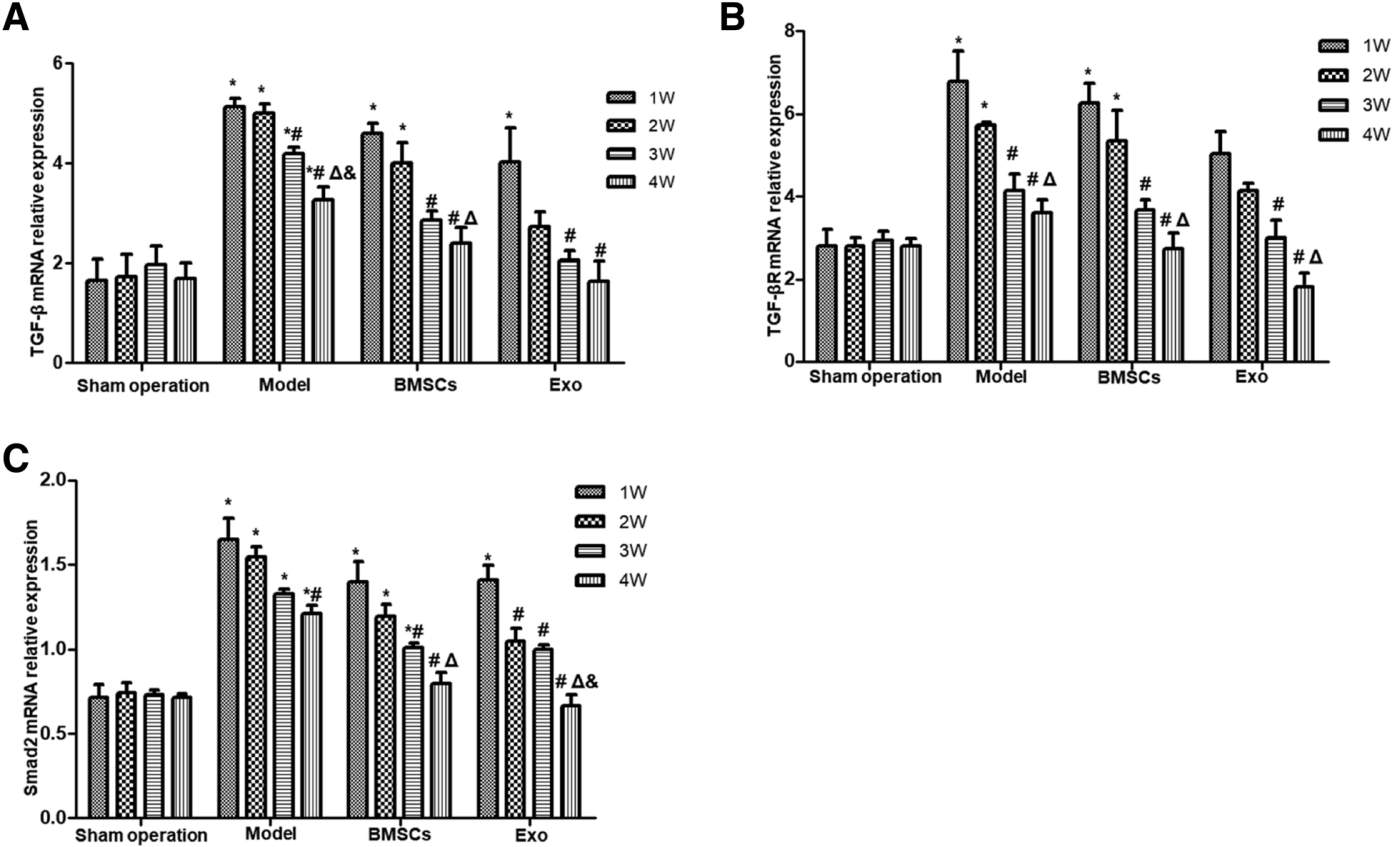
The relative mRNA expression of TGF-β1, TGF-βR1, and Smad2 in rabbit endometrium. **a** The relative mRNA expression of TGF-β1; **b** The relative mRNA expression of TGF-βR1; **c** The relative mRNA expression of Smad2. *P < 0.05, compared to the Sham operation group; ^#^*P* < 0.05, compared with 1 week within the same group; ^Δ^*P* < 0.05, compared with 2 weeks within the same group; ^&^*P* < 0.05, compared with 3 weeks within the same group

RT-PCR was performed to detect the expression of TGF-β1, TGF-β1R, and Smad2 mRNA in the endometrium after injury and treatment (Fig. 7). Compared with the Sham operation group, the expression levels of TGF-β1 (Fig. 7a), TGF-β1R (Fig. 7b), and Smad2 mRNA (Fig. 7c) significantly increased after endometrial damage (*P* < 0.05). The expression of TGF-β1, TGF-β1R, and Smad2 mRNA in the BMSC group and Exo group decreased significantly with the prolongation of treatment time (*P* < 0.05). The expression of TGF-β1, TGF-β1R, and Smad2 mRNA decreased in the model group with the prolonged treatment time. After 3 weeks of treatment, the relative expression of TGF-β1 mRNA in the BMSC group had no significant difference from the Sham operation group (*P* > 0.05). After 2 weeks of treatment by Exo, there was no significant difference between the Exo group and the Sham operation group in the expression of TGF-β1 mRNA (*P* > 0.05). Three weeks after treatment, the relative expression of TGF-β1R mRNA between the model group, BMSC group, and Exo group had no significant difference compared with the Sham operation group (*P* > 0.05). After 4 weeks of treatment, the relative expression of Smad2 mRNA in the BMSC group had no significant difference from the Sham operation group (*P* > 0.05). After 2 weeks of treatment, there was no significant difference between the Exo group and the Sham operation group (*P* > 0.05). These suggest that endometrial damage is accompanied by the upregulation of TGF-β1, TGF-β1R, and Smad2 mRNA, and the treatment with Exo and BMSCs can downregulate the expression of TGF-β1 and Smad2 mRNA.

### The effect of BMSC and Exo treatment on the protein phosphorylation of TGF-β1, TGF-β1R, and Smad2 of the endometrial damaged uterus

**Fig. 8:**
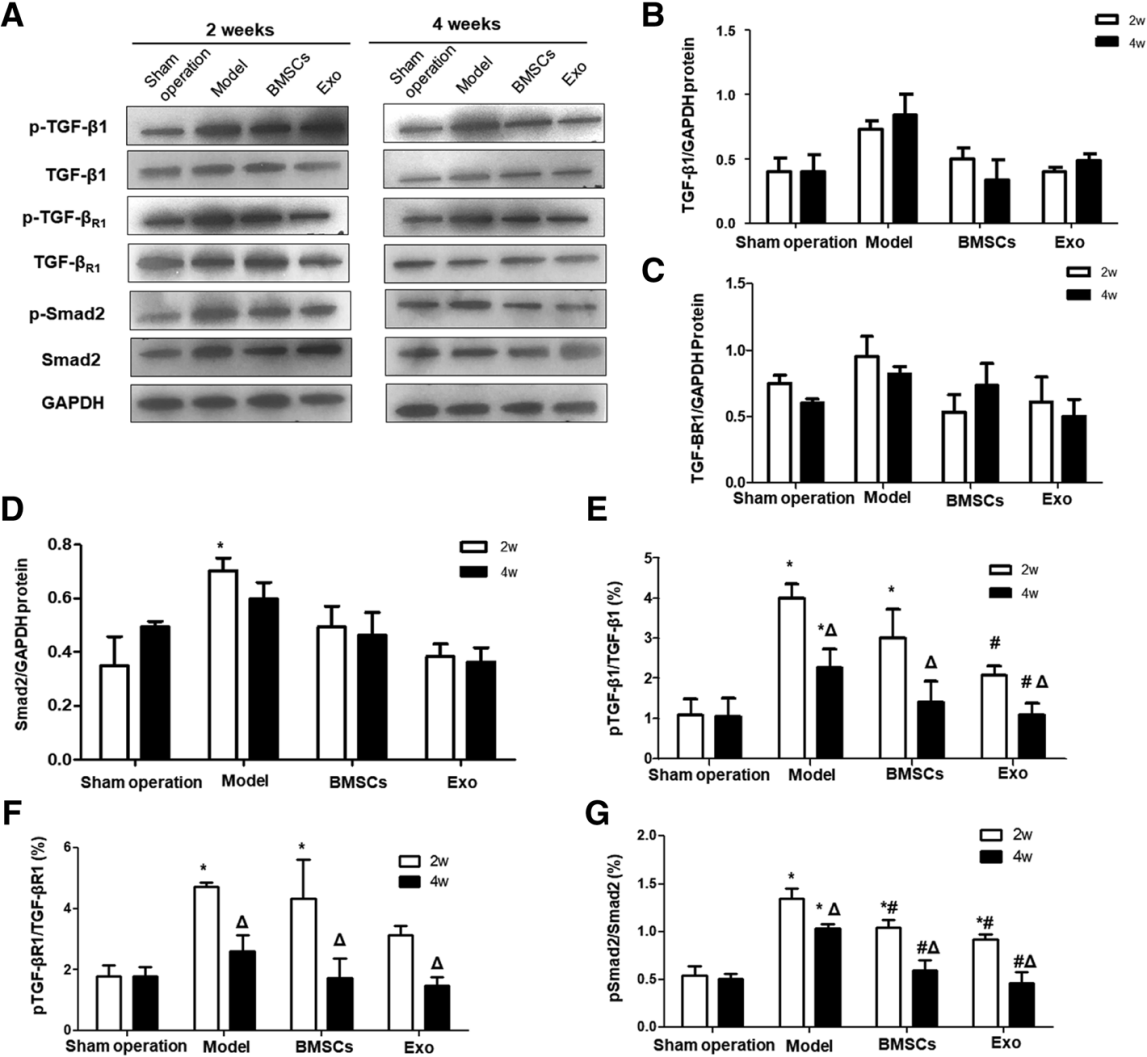
The expression of phosphorylated TGF-β1, TGF-βR1 and Smad2 in rabbit endometrium. **a** The expression of TGF-β1, TGF-βR1, and Smad2 and their phosphorylated forms detected by Western blot at different time points. The protein expressions of **b** TGF-β1, **c** TGF-βR1, and **d** Smad2 were quantified in relative to that of GAPDH. **e** The percentage of p-TGF-β1 in the total TGF-β1 protein. **f** The percentage of p- TGF-βR1 in the total TGF-βR1 protein. **g** The percentage of p-Smad2 in the total Smad2 protein. *P < 0.05, compared to the Sham operation group; ^#^*P* < 0.05 compared to the model group; ^Δ^*P* < 0.05, comparison between the expressions at 2 weeks and 4 weeks

Western blot was performed to detect the protein phosphorylation of TGF-β1, TGF-β1R, and Smad2 in the endometrium after injury and treatment (Fig. 8). After endometrial injury, the phosphorylation levels of TGF-β1, TGF-β1R, and Smad2 were significantly increased (*P* < 0.05). With the prolongation of treatment time, the phosphorylation levels of TGF-β1, TGF-β1R, and Smad2 protein in the model group decreased, but there was no significant difference compared to that of the Sham operation group (*P* > 0.05). The phosphorylation levels of TGF-β1, TGF-β1R, and Smad2 in the BMSC group were not significantly different from those in the Sham operation group at 4 weeks after treatment (*P* > 0.05). For the Exo group, the phosphorylation levels of TGF-β1 and TGF-β1R were not significantly different from those in the Sham operation group after 2 weeks of treatment (*P* > 0.05), and the phosphorylation level of Smad2 protein had no significant difference from that of the Sham operation group after 4 weeks of treatment (*P* > 0.05). These suggest that BMSC and Exo treatment significantly downregulate the phosphorylation levels of TGF-β1, TGF-β1R, and Smad2 protein after uterine injury.

### Culture of EECs

**Fig. 9:**
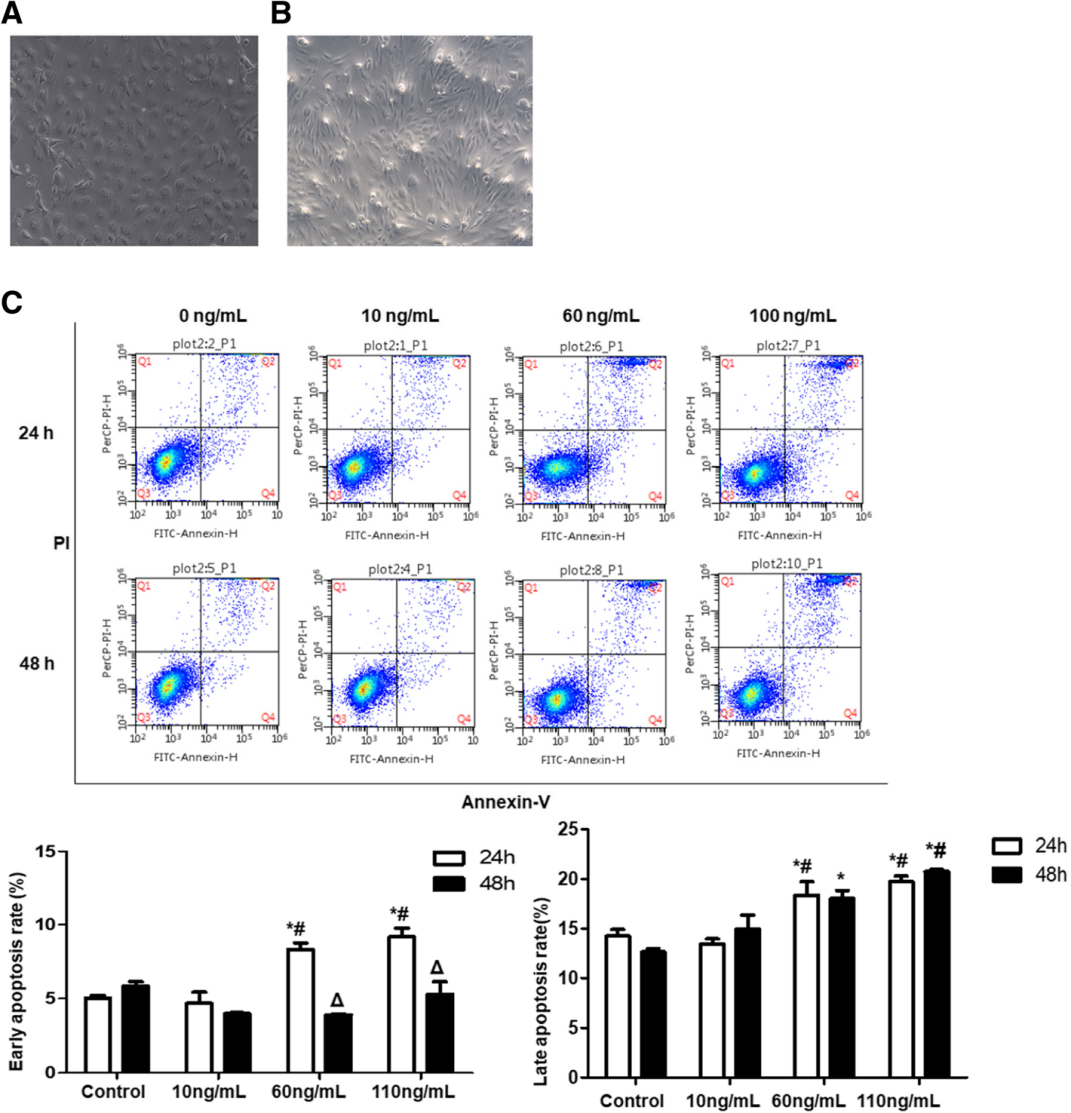
Endometrial epithelial cell morphology, immunohistochemical identification, and post-induction morphological changes. **a** Morphology of P1 generation endometrial epithelial cell (× 100). **b** Morphology of P2 generation endometrial epithelial cell after treatment with 60 ng/ml TGF-β1 for 24 h (× 100). **c** Comparison of apoptotic rates between groups under intervention of different concentrations of TGF-β1.*P < 0.05, compared to control group at the same time point; ^#^*P* < 0.05, compared to 10 ng/mL at the same time point; ^Δ^*P* < 0.05, compared between 24 h and 48 h at the same concentration

The cell morphology of EECs was observed. Cells began to adhere to culture flask on D3 of inoculation and some EECs adhered to culture flask after the first 24 h of inoculation in the shape of irregular triangles. On D14, cells reached full confluency (Fig. 9a). The cells were in circular or elliptical shapes, with clear cell boundaries and full cytoplasm. Nucleus located in the center of the cell. Cells were arranged closely, like paving stone.

### EMT under the induction of TGF-β1 on endometrial epithelial cell

After incubation with TGF-β1 for 24 h or 48 h, EECs were observed under inverted microscope. It is found that EECs were elongated or had filamentous pseudopods. Cells were in spindle shape, with blurred boundaries and enlarged cell gap. Cell density decreased, especially under the induction of 60 ng/ml TGFβ1 for 24 h, and most cells became fusiform, and cell gap was remarkably increased (Fig. 9b).

Flow cytometry was used to detect the apoptosis of EECs. As shown in Fig. 9c, after treatment with 60 ng/mL or 110 ng/mL TGF-β1 for 24 h, the early and late apoptosis rates of EECs were significantly increased than the control group (*P* < 0.05). After treatment with 60 ng/mL or 110 ng/mL TGF-β1 for 48 h, the late apoptosis rate was significantly increased than control (*P* < 0.05). The early apoptosis rate after treatment with TGF-β 1 (60 ng/mL and 110 ng/ml) for 48 h was significantly higher than that for 24 h. It is shown that 60 ng/mL TGF-β1 can significantly induce EEC apoptosis after 24 h stimulation. Thus, the optimal induction concentration and time of TGF-β1 was determined as 60 ng/ml TGFβ1 for 24 h.

### Exo derived from BMSC reverse EMT of EEC

**Fig. 10:**
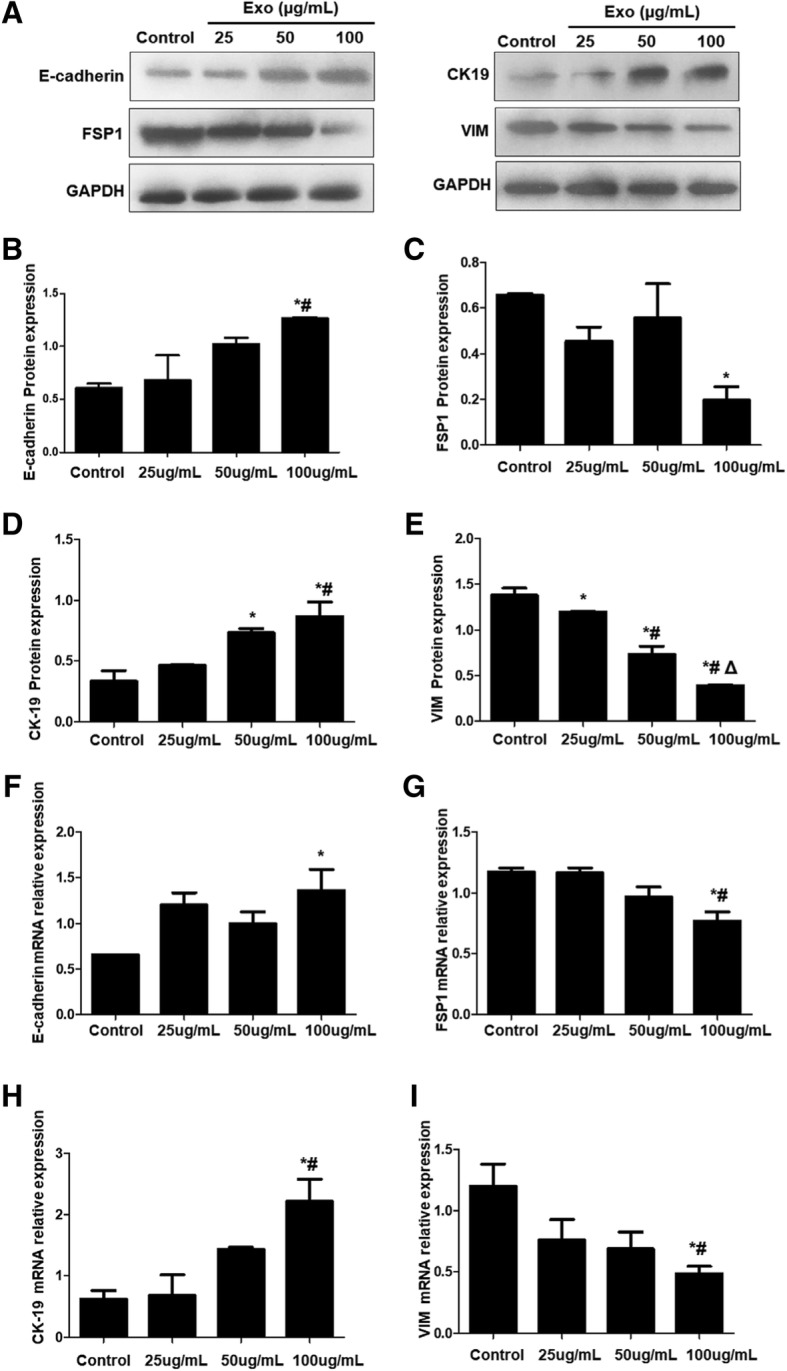
Expression changes of EMT-related proteins and mRNAs. The expression of E-cadherin, FSP1, CK19, and VIM at protein and mRNA levels were analyzed. **a** Representative Western blot results. **b**–**e** Quantitative Western blot results of E-cadherin, FSP1, CK19 and VIM, respectively. **f**–**i** mRNA levels of E-cadherin, FSP1, CK19, and VIM, respectively. *P < 0.05, compared with control; ^#^*P* < 0.05, compared with 25 μg/mL Exo; ^Δ^*P* < 0.05, compared with 50 μg/mL Exo

EECs were first treated with 60 ng/ml of TGF-β1 for 24 h to induce EMT and then with various concentrations of Exo for 24 h. Western blot was used to detect the expression level of E-cadherin, FSP1, CK19, and VIM. Results showed that compared with the control group, the expression of E-cadherin was significantly increased (*P* < 0.05) at 100 μg/ml of Exo (Fig. 10a, b). At 25 μg/ml and 50 μg/ml, expression of E-cadherin was slightly increased but without significant difference compared with the control group (*P* > 0.05). Expression of FSP1 was significantly decreased at 100 μg/ml (*P* < 0.05) compared with control group (Fig. 10a and c). Compared with control group, expression of CK19, the endometrial specific marker protein, was significantly increased at 50 μg/ml and 100 μg/ml (*P* < 0.05) (Fig. 10a, d) while the expression of VIM was significantly decreased at 25 μg/ml, 50 μg/ml, and 100 μg/ml (*P* < 0.05) (Fig. 10a, e).

RT-PCR was used to detect mRNA expression changes of E-cadherin, FSP1, CK19, and VIM. Results showed that at 100 μg/ml of Exo, the expression of CK19 (Fig. 10f) and E-cadherin mRNA (Fig. 10g) were significantly increased (*P* < 0.05) while expression of VIM (Fig. 10h) and FSP1 (Fig. 10i) were significantly declined (*P* < 0.05). It is indicated that BMSC-derived Exo may effectively reverse endometrial EMT induced by TGF-β1.

**Fig. 11:**
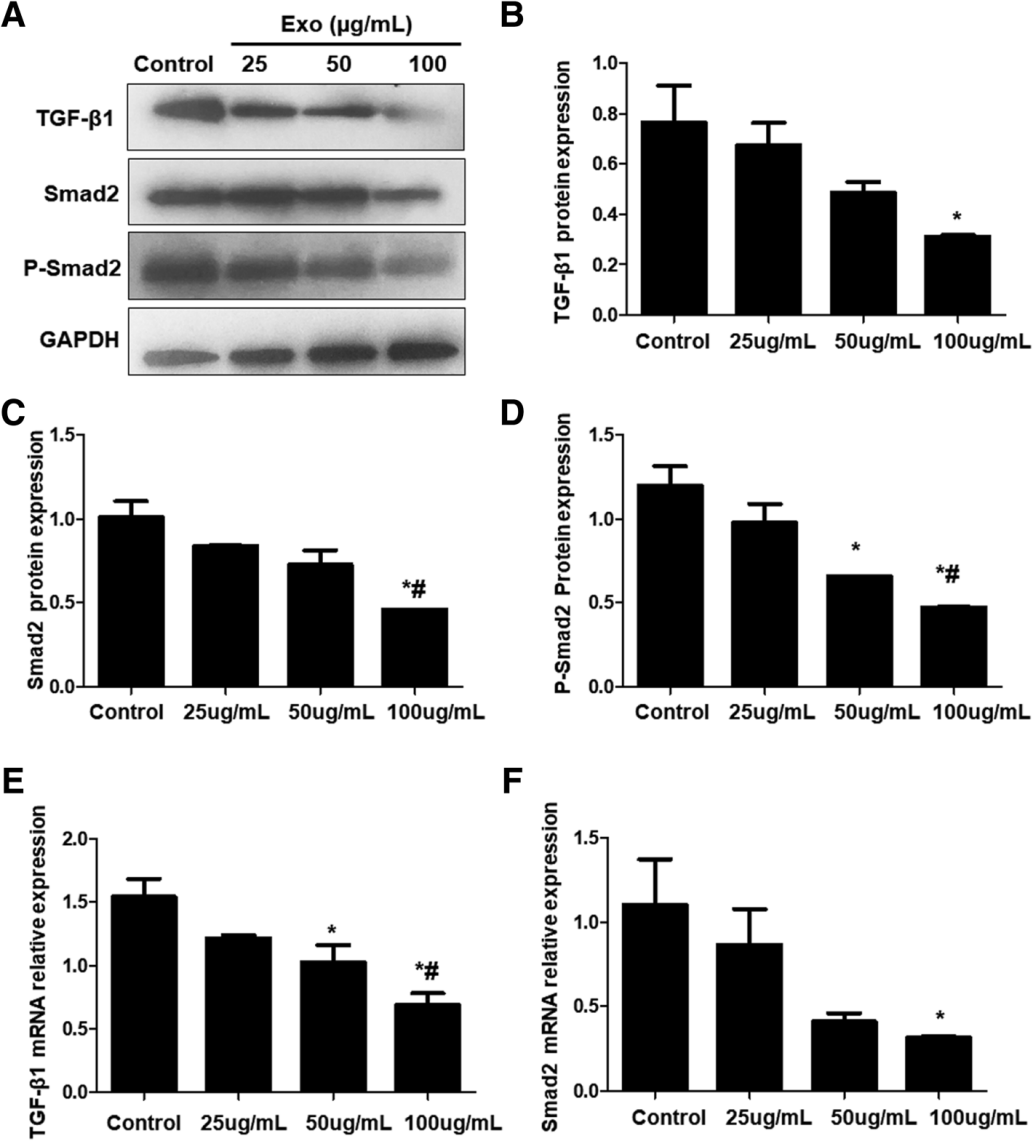
Expression changes of TGF-β/Smad signaling pathway-related factors. Western blot and qRT-PCR was used to detect the protein and mRNA expression of TGF-β, Smad2, and p-Smad2. **a** Representative Western blot results. **b**–**d** Quantitative Western blot results of TGF-β1, Smad2, and p-Smad2, respectively. **e** and **f** mRNA levels of TGF-β and Smad2 by qRT-PCR. *P < 0.05, compared with control; ^#^*P* < 0.05, compared with 25 μg/mL Exo

Western blot was used to detect expression changes of TGF-β 1 and Smad 2 protein in TGF-β1/Smad signaling pathway during EMT. Results showed that with the increase of Exo concentration, protein expression of TGF-β1, Smad 2, and P-Smad2 gradually decreased, which was significantly lower than that of the control group when Exo was 100 μg/ml (*P* < 0.05) (Fig. 11a–d). Expression of p-Smad 2 significantly decreased when Exo was 50 μg/ml (*P* < 0.05), which was lower than the control group (Fig. 11a–d).

RT-PCR was used to detect expression changes of TGF-β1 and Smad2 mRNA. Results showed that expression of both TGF-β 1and Smad2 mRNA gradually decreased with the increase of Exo concentration. Comparing with the control group, expression of TGF-β1 mRNA was significantly decreased when the concentration of Exo reached 50 μg/ml (*P* < 0.05), and expression of Smad2 mRNA was significantly decreased when Exo concentration reached 100 μg/ml (*P* < 0.05). It is indicated that BMSC-derived Exo may reverse EMT by regulating TGF-β/Smad signaling pathway.

## Discussion

At present, the main treatment for IUA is surgery combined with estrogen therapy. However, the recurrence rate is as high as 20–63% [14]. Moreover, the placental implantation risk will also be increased [15]. Thus, it is urgent to develop a safe and effective treatment for IUA patients.

EMT plays a major role in most fibrotic diseases [2, 16]. Bai Y et al. [17] found that resveratrol inhibited EMT of renal tubules and extracellular matrix by antagonizing Hedgehog signaling pathway in vitro and in vivo, which reduced expression of TGF-β1 and suppressed renal fibrosis. Bi WR et al. [18] proposed that bone morphogenetic protein-7 could induce down-regulation of 1α-SMA mRNA and up-regulation of E-cadherin mRNA, which could promote the transformation of EMT to mesenchymal-epithelial transition (MET) in rat hepatocyte, thereby reducing liver fibrosis. Sun et al. [19] confirmed that miRNA-29b could inhibit EMT induced by silica in RLE-6TN cells and promote MET. More and more researchers believe that endometrial EMT may be one of the main mechanisms of IUA [3–5]. Thus, inhibiting EMT in EEC may be a new strategy for the treatment of IUA.

MSCs are widely used in the repair of injured tissues. Gargett et al. [20] proposed that endometrial MSCs could be used to rebuild endometrial tissue in patients with IUA. Nagori et al. [21] found that transplantation of BMSCs in vivo could promote endometrial repair after injury. Later, Phermthai et al. [22], Du et al. [8], and Cao et al. [23] reported that MSC transplantation could effectively repair endometrial defects including endometrial hyperplasia and infertility. MSCs also play an anti-fibrotic role in fibrotic diseases such as liver fibrosis [24, 25], kidney fibrosis [26], and lung fibrosis [27, 28]. However, colonization differentiation rate of MSCs in host tissues is very low [20]. In recent years, studies [29, 30] have found that Exo could deliver functional proteins, RNA and miRNA between cells. MSCs may play its biological roles in repairing injured tissue by secreting Exo. It is shown that MSC-derived microvesicles can inhibit EMT of nephrocyte through miRNA regulating signaling pathways, which could effectively improve renal interstitial fibrosis [31]; in hepatic fibrosis model induced by carbon tetrachloride, transplantation of MSCs-derived Exo could inhibit EMT in hepatocyte, alleviate inflammation, reduce collagen deposition, and regulate fibrosis-related signaling pathways, thus improving liver fibrosis [32]. It is suggested that MSC-derived Exo might play a role in fibrotic diseases by reversing EMT. Based on these findings, we propose that BMSC-derived Exo could be used in IUA treatment.

In our rabbit model of IUA, we found that compared to the Sham operation group, the number of endometrial glands in the model group was significantly decreased, and the endometrial fibrosis area increased. The expression of CK-19 was significantly downregulated while the expression of VIM was significantly up-regulated, which suggested that endometrial epithelial converted to mesenchyme in the process of fibrosis repair of injured endometrium; in the treatment group of BMSCs and Exo, it was found that compared to model group, with the increase of treatment time, the number of endometrial glands significantly increased while the area of endometrial fibrosis decreased, the expression of CK19 was significantly up-regulated while the expression of VIM was significantly downregulated, and there was no significant difference in the effects of BMSCs and Exo. Thus, BMSCs and Exo could promote endometrial repair, thus inhibiting uterine fibrosis and reducing scar repair. However, Exo is superior to BMSCs in terms of timeliness. Thus, we suppose that Exo may be the main functional factor in the injury repairing of BMSCs.

EMT is a complicated biological process which may involve one or more signaling pathways. There is a close relationship between TGF-β/Smad signaling pathway and endometrial fibrosis [4, 5]. Zhu et al. [33] also described Hippo and Wnt signaling pathway could form a complex signaling pathway network with TGF-β signaling pathway to mediate endometrial fibrosis. It was found in this study that with the increase of treatment time, the expression of TGF-β1, TGF-β1R, and Smad2 mRNA in the model group was significantly higher than that in the Sham operation group. While in BMSC and Exo treatment groups, the mRNA and protein expressions of TGF-β1 and Smad2 were significantly downregulated compared to model group, and Smad2 phosphorylation level was also significantly reduced, suggesting that endometrial fibrosis repair may be related to TGF-β1/Smad signaling pathway and that MSC-derived Exo may regulate fibrosis repair of injured endometrium by downregulating TGF-β1/Smad signaling pathway.

TGF-β1 is one of the main factors in inducing EMT. It could promote aggregation of fibroblasts and inflammatory cells, as well as the synthesis of collagen and fibrin, which further results in extracellular matrix deposition and degradation, thus promoting cell differentiation and apoptosis. Therefore, it is widely used for EMT induction in vitro. EMT has been found in many tissues/cells, such as bronchial epithelial cells [34], renal tubular epithelial cells [35], intestinal epithelial cells [36], and retinal epithelial cells [37]. All of these cells could undergo EMT under the induction of TGF-β1. First, TGF-β binds to the TGF-β receptor on the cell membrane to form a ligand-receptor complex, which in turn binds and phosphorylates Smad protein family to form a complex. Then, the complex enters the nucleus and interacts with transcription factors to regulate downstream gene transcription related to EMT, and ultimately EMT is induced. In our study, rabbit EECs were treated with different concentrations of TGF-β1 for 24 h and 48 h. Under microscopy, degree of cell morphology transformation was found to be positively correlated with the concentration and duration of TGF-β1. Cell apoptosis was increased with the increase of TGF-β1 concentration. Comparing with the control group, apoptotic rate were both statistically significant in 60 ng/ml and 100 ng/m dose groups inducing for 24 h and 48 h. Except for the group of 60 ng/ml inducing for 24 h, the degree of apoptosis of EECs was much higher than that of the control group, which suggested that 60 ng/ml TGF-β1 inducing uterine epithelial cells for 24 h was less cytotoxic.

To analyze the effect of Exo on EMT, we first treated EECs with TGF-β1 and then with Exo. CK-19, VIM, FSP-1, and E-cadherin were selected as indicators of detecting EMT changes. CK-19 is a specific marker of epithelial cells and VIM is a specific marker of mesenchymal cells, which are considered as active factors of EMT. FSP-1 is a type 2 EMT marker for fibrosis; E-cadherin is a transmembrane protein expressed in almost all epithelial cells, mediating adhesion between cells of the same type and playing an important role in maintaining the stability of epithelial cells. The downregulation of E-cadherin is the first and the most important step of EMT. As the increase of Exo concentration, protein and mRNA expression of CK-19 and E-cadherin tended to increase, and expression of VIM, FSP-1, TGF-β1, Smad 2, and P-Smad 2 tended to decline, which suggests that after administration of Exo, the cell phenotype converts to epithelial cell phenotype, intercellular adhesion is enhanced, and expression of EMT marker is decreased. BMSC-derived Exo could reverse EMT in EEC via the TGF-β1/Smad signaling pathway.

Valadi et al. [38] proposed that mRNA and microRNAs contained in Exo could transmit between cells and act as Exosomal shuttle RNA, and a large number of studies [39–41] reported that miRNA inhibited EMT by mediating EMT-related signaling pathways. In summary, we speculate that the reason of BMSC-derived Exo being able to reverse EMT in endometrial epithelial cells may be related with its active substances, which directly or indirectly mediated TGF-β1/Smad signaling pathway. The specific mechanism still stays unclear and it needs to be further investigated. There is lipid raft distributing on the surface of MSCs-derived Exo, which favors protein-protein interaction and conformation transformation in signal transduction and intercellular protein transport and may be used as a carrier for targeted therapy [42–44].

For the first time, BMSC-derived Exo was reported to be able to reverse EMT in endometrial glandular epithelial cells in our study. However, it needs to be further investigated on which active substance in BMSC-derived Exo or which adhesion molecule on its surface plays a key role in this process.

## Conclusion

In our study, BMSC-derived Exo may regulate repair of injured endometrium by TGF-β1/Smad signaling pathway. The tissue regeneration and repair function of BMSCs may work via its paracrine effect rather than its multi-directional differentiation potential. Early intervention with BMSC-derived Exo could reverse EMT in rabbit EECs, which suggests that reversing EMT in EECs with Exo derived from BMSCs may provide new ideas for prevention and treatment of IUA.

## Data Availability

All data generated or analyzed during this study are included in this published article [and its supplementary information files].

## Abbreviations

BMSCs: Bone marrow mesenchymal stem cells
CK-19: Cytokeratin-19
EECs: Endometrial epithelial cells
EMT: Epithelial-mesenchymal transition
FSP-1: Fibroblast-specific protein-1
HSP70: Heat shock protein 70
IUA: Intrauterine adhesion
MET: Mesenchymal-epithelial transition
MSC: Mesenchymal stem cell
TGF: Transforming growth factor
VIM: Vimentin
